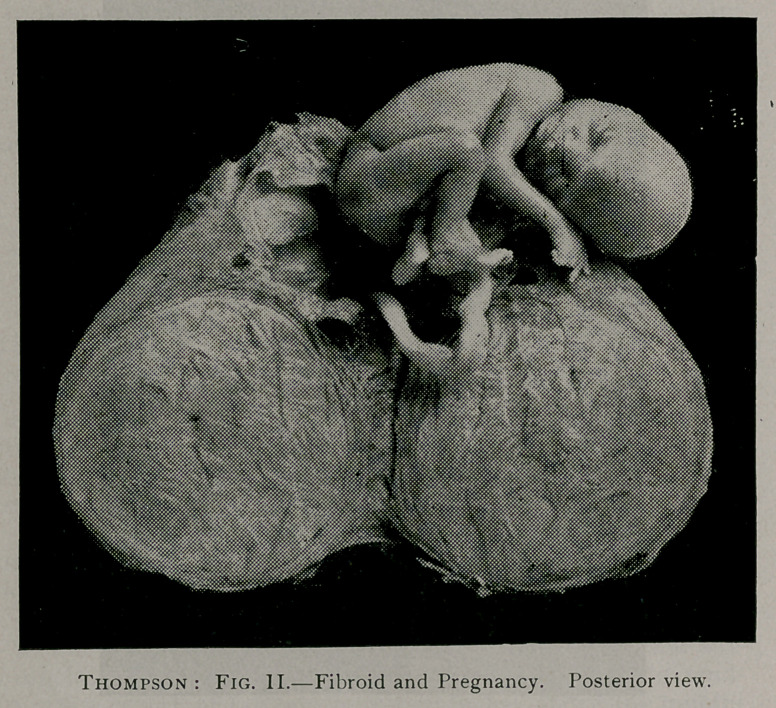# Fibroid Tumor Complicating Gestation—Hysterectomy—Recovery

**Published:** 1905-02

**Authors:** F. D. Thompson

**Affiliations:** Fort Worth, Texas. Professor of Gynecology and Surgical Diseases of Women, Medical Department, Fort Worth University


### Fibroid Tumor Complicating Gestation—Hysterectomy—Recovery.

By F. D. THOMPSON, M. D., Fort Worth, Texas.
Professor of Gynecology and Surgical Diseases of Women, Medical Department, Fort Worth University.

[From *The Texas Medical Gazette*, June, 1904.]

I THINK the following case is of sufficient interest to the profession to justify me in reporting it:

On Thursday, April 14, 1904, Mrs. L., white, American, age 33, married twelve years, no children, no miscarriages, complexion fair and general health good, came to me from Canton, Ga., on account of an abdominal tumor which was growing rapidly. During the year 1903 her menstrual flow was increased in duration and quantity. In the early part of December, 1903, her flow ceased entirely and has not appeared since. In January, 1904, the patient observed a tumor just above the pubis and a little to the left side, and during February and March she suffered much with nausea. On examination I found a tumor extending from the cervix of the uterus to two inches above the umbilicus. The lower half of the tumor was very hard and firm; the upper half was soft as compared with the lower portion. The cervix, which was not involved, seemed to be attached to the center of the hard part of the tumor. As much of the tumor seemed to be in front of the cervix as behind it.

With this history and condition, I made the diagnosis of a fibroma occupying the lower portion of the uterus complicated with a four months' pregnancy in upper portion of the uterus. I advised an operation, consisting of the removal of the uterus and the appendages, as giving the patient the best chance for her life. This was consented to and on Saturday morning, April 16, 1904, I did an abdominal hysterectomy, removing the uterus, ovaries and tubes. The operation was performed as follows: the abdomen was opened freely, the incision extending an inch or more above the umbilicus; the uterus was lifted out of the abdomen; the ovarian artery on the left side was ligated; the clamp forceps placed next to the uterus; the artery and broad ligament were cut between the clamp and the ligature down to the round ligament. This was ligated and a clamp placed next to the uterus; the round ligament was divided and the broad ligament cut down

THOMPSON: FIG. I.—Fibroid and Pregnancy. Anterior view.

to the uterine artery at the junction of the cervix with the tumor. The folds of the broad ligament were slightly separated; the uterine artery was ligated by passing a threaded aneurism needle under it; a clamp was applied next to the uterus and the uterine artery was then divided. The peritoneum from the broad ligament on the left side extending to the broad ligament on the right side and about half an inch above the bladder in front, was divided with the knife. The peritoneum and bladder were dissected down slightly below the junction of the cervix with the body of the uterus. The peritoneum was divided in the same way on the posterior surface of the uterus and tumor and pushed down to the cervix, then the cervix was separated from the uterus and tumor. When it was cut through, the tumor was lying well over to the right side, the right uterine artery could be seen and felt between the folds of the broad ligament. This was ligated, clamped, and divided. The broad ligament was then divided up to the round ligament. It was ligated, clamped, and divided. When the broad ligament was divided to the right ovarian artery, this was ligated and cut including the infundibulo-pelvic liga-

THOMPSON: FIG. II.—Fibroid and Pregnancy. Posterior view.

ment. This completed the separation of the uterus, ovaries, and tubes from the patient. The ovarian and uterine arteries were re-tied for safety. I then closed the stump of the cervix (which had been hollowed out during its amputation) with ten-day catgut sutures. I next began with a continuous catgut suture to unite folds of the broad ligament by starting where I put the first ligature on the left ovarian artery, continuing down to the cervix and turning in the ligated ends to the arteries. When I reached the cervix I closed the peritoneum over it, continuing in the same way until I reached the ovarian artery on the right side. When this was done the pelvis was absolutely clean showing only the continuous suture that had brought the edges of the broad ligament together. The abdomen was closed in the usual way and the patient put to bed in good condition.

I then opened the fundus of the uterus and exposed the four and a half months' fetus. The wall of the uterus when opened was not more than an eighth of an inch thick. So much of the uterus was involved in the fibroma that I believe the uterus would have ruptured long before the full term of gestation was reached.

The patient made an uninterrupted recovery, leaving the infirmary apparently well four weeks after the operation. The cuts indicate the position of the fibroid and fetus.

## Figures and Tables

**Fig. I. f1:**
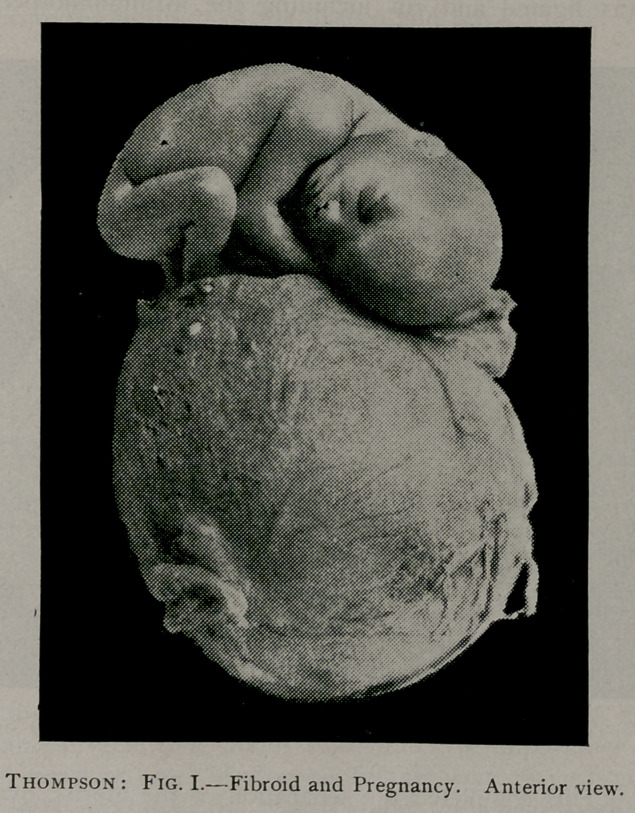


**Fig. II. f2:**